# Gliomatosis Cerebri Manifested as Parkinsonism Syndrome: A Case Report

**DOI:** 10.1155/crnm/3375867

**Published:** 2024-11-28

**Authors:** Debora González Garcia, Miguel Ángel González Casas, Martin Heisi Gómez Martínez

**Affiliations:** ^1^Department of Internal Medicine, Regional General Hospital No. 1 of Queretaro, Queretaro, Mexico; ^2^Neurology Department, Institute of Security and Social Services for State Workers, Saltillo, Coahuila, Mexico; ^3^Neurosurgery Department, Institute of Security and Social Services for State Workers, Saltillo, Coahuila, Mexico

**Keywords:** case report, central nervous system, diffuse astrocytoma, gliomatosis cerebri, parkinsonism

## Abstract

**Introduction:** Gliomatosis cerebri (GC) is a diffuse neoplastic process, whose presentation is extremely rare and lacks a characteristic clinical pattern. The objective of this case is to describe the clinical aspects of a patient with GC, in whom symptoms of parkinsonism and neurocognitive issues predominate.

**Case Report:** A 78-year-old patient with no significant medical history was referred to the neurology consultation due to balance disturbances accompanied by head tremor. Symptoms of parkinsonism progressively worsened, adding cognitive and neuropsychiatric disorders. Cranial magnetic resonance imaging (MRI) showed diffuse and generalized white matter hyperintensity. Under the suspicion of GC, a frontal lobe biopsy was performed, with a pathology report of diffuse astrocytoma, thus confirming the diagnosis of GC.

**Conclusion:** GC is a disease that presents with nonspecific clinical manifestations, making a clinical diagnosis challenging. It should be suspected in cases of parkinsonism accompanied by other focal neurological disorders. This leads to delayed diagnosis and consequently low incidence. The importance of MRI as a diagnostic aid is highlighted, with biopsy being necessary to confirm the diagnosis.

## 1. Introduction

Gliomatosis cerebri (GC) is a diffuse neoplastic process, whose presentation is extremely rare and lacks a characteristic clinical pattern. The term was first proposed in 1938 by Nevin. Today, the World Health Organization (WHO) defines it as a pattern of widespread infiltrative growth affecting at least three cerebral lobes bilaterally, often extending to the brainstem, cerebellum, and even the spinal cord [1, 2]. Most histologically correspond to Grade II, III, or IV astrocytomas, which have a worse prognosis compared to other gliomas of the same grade. According to the Surveillance, Epidemiology, and End Results (SEER) database network in the United States (1973–2012), the estimated incidence is about 0.10 per million individuals, with a peak incidence after 65 years of age of 0.43 per million, with a male predilection [3]. Although the suspected diagnosis is based on clinical presentation and neuroimaging findings, it is confirmed through a histopathological study. Patients may present with progressive headaches, neurocognitive and personality disorders, and symptoms mimicking dementia.

We present a case report of GC with an unusual presentation in a 78-year-old patient debuting with parkinsonism syndrome.

## 2. Case Presentation

**Figure 1:**
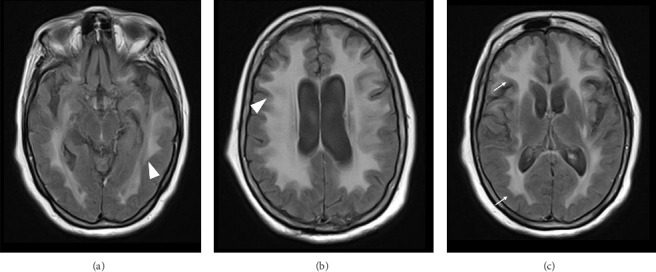
MRI of the patient's brain in the T2/FLAIR sequence, generalized white matter involvement is observed. (a) A slice at the midbrain level shows white matter involvement in the temporal lobes. (b) The section at the lateral ventricles level shows generalized hyperintensity with extension into the overlying cortex. (c) A slice at the level of the insula and thalamus shows white matter involvement in the frontal and parietal lobes.

**Figure 2:**
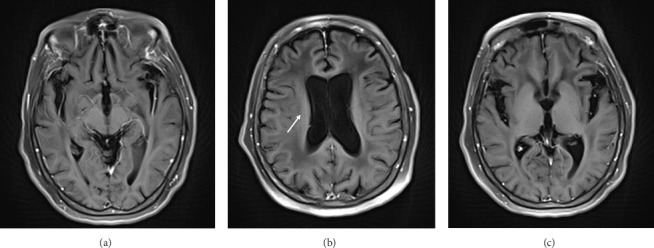
Transaxial brain sections in T1 with application of contrast medium show no enhancement in the cerebral lobes. (a) Slice at the midbrain level shows white matter involvement in the temporal lobes without enhancement with contrast agent. (b) Slice at the lateral ventricles level shows white matter involvement in the semioval centers without contrast agent uptake. (c) The section at the level of the insula and thalamus without contrast enhancement.

**Figure 3:**
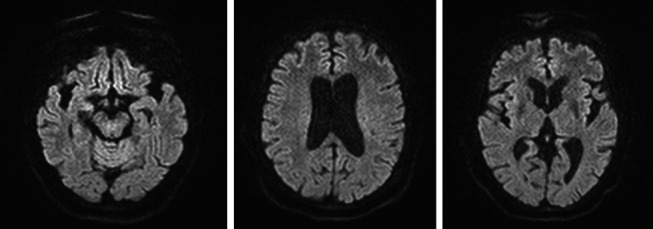
DWI sequence with various slices shows no diffusion restriction.

A 78-year-old Mexican woman with no relevant family or personal medical history presented in 2018 with gait disturbances such as imbalance and slowness of walking, leading to repeated falls, accompanied by head tremor. A year later, she developed recent memory impairment and sporadic psychotic episodes. By 2022, her condition had progressed to greater walking disability, marked rigid-akinetic syndrome, requiring support for ambulation, and eventually becoming wheelchair-bound. Nonmotor symptoms included limited speech, reduced comprehension, emotional liability, and dependency on daily living activities. During that year, a brain MRI showed diffuse, generalized hyperintensity of the supratentorial white matter in T2/FLAIR sequences across all cerebral lobes (Figure 1). Unlike the T1 sequence, where no contrast enhancement was observed (Figure 2), and the DWI sequence, where there was no diffusion restriction (Figure 3), ruling out other possible differential scenarios. Cerebrospinal fluid (CSF) analysis revealed mildly elevated protein levels at 56.6 mg/dL (normal range: 15–45 mg/dL), with normal cell count and glucose levels, of 0/mm3 and 59 mg/dL respectively. Tests for KOH, bacterioscopy, culture, cytology, and JC virus in the CSF were negative.

**Figure 4:**
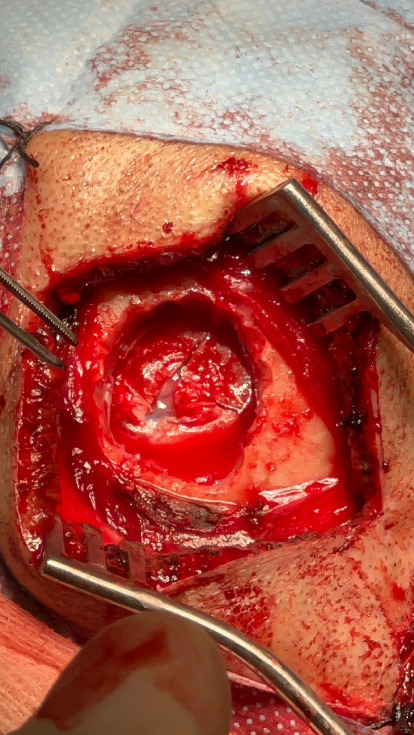
Craniotomy performed for a frontal lobe biopsy.

**Figure 5:**
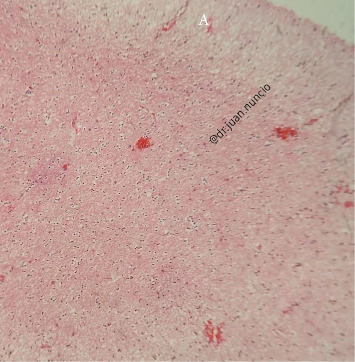
Histological section of brain biopsy with hematoxylin and eosin staining. Diffuse astrocytoma Grade II. The exterior surface is gray-red, dome-shaped with a smooth appearance, bordered by fibrillary walls surrounded by sparse superficial erythematous stroma.

Given the clinical presentation and ruling out infectious pathology through the CSF analysis, combined with radiological findings, GC was highly suspected. A cerebral biopsy via neuroendoscopic surgery was performed (Figure 4), revealing a pathology report of diffuse astrocytoma, confirming GC (Figure 5).

In May 2022, a diagnosis of GC was concluded. Currently, the patient shows moderate cognitive deterioration, with improvement in parkinsonism symptoms but still requires support for ambulation and experiences intermittent neuropsychiatric changes. She is being treated with good results with levodopa-carbidopa, antipsychotic olanzapine, and antidepressant escitalopram.

## 3. Discussion

GC was first described as excessive and diffuse growth of neoplastic cells in areas of both cerebral hemispheres. The WHO currently defines it as “a diffuse glioma, usually astrocytic, with a growth pattern consisting of exceptionally extensive infiltration of a large region of the central nervous system (CNS), involving at least three cerebral lobes, usually bilaterally affecting the cerebral hemispheres and/or gray matter, often extending to the brainstem, cerebellum, and even the spinal cord” [1, 4].

### 3.1. Epidemiology

While there is a broad range of presentation from 17 to 85 years of age, it peaks between 40 and 50 years old [5], being more common in men than in women with a ratio of 1,3:1 [1, 6, 7].

### 3.2. Classification

It is typically classified into Type I and Type II. Type I GC is the classic form with diffuse infiltration of neoplastic glial cells but without a tumor mass. Type II involves diffuse infiltration along with a tumor mass. Secondary GC is defined as infiltrative dissemination of tumor cells from a previously diagnosed glioma and is associated with prior radiation or antiangiogenic therapy [8–10]. GC generally exhibits aggressive biological behavior, classified by the WHO as Grade III malignancy. Even if biopsy shows cellular anaplasia, it is still considered high-grade malignancy [6].

### 3.3. Clinical Manifestations

The clinical presentation depends on the affected area. There are no typical signs or symptoms of GC due to the unpredictable invasion of tumor cells. However, the most common manifestations include seizures and focal neurological signs [5]. Patients may present with progressive headaches, neurocognitive and personality disorders, and symptoms mimicking dementia. Infratentorial involvement can present with gait disturbances, ataxia, cerebellar signs, cranial nerve palsies, and parkinsonism symptoms when the basal ganglia are affected [1, 10]. This was the case with our patient, who presented predominantly with rigid-akinetic syndrome, consistent with basal ganglia involvement observed in neuroimaging.

Magnetic resonance imaging (MRI) has greater sensitivity, showing diffuse hyperintensities in T2/FLAIR sequences, predominantly in white matter, with loss of gray-white differentiation, cortical thickening, and corpus callosum involvement, and a ventricular horn collapse is suggestive of GC. Due to the low specificity of MRI, histological confirmation is mandatory [1].

Radiologically, it is difficult to distinguish GC from other pathologies. Hyperintensities in T2 can be observed in the white matter in demyelinating diseases, including progressive multifocal leukoencephalopathy and cerebral vasculitis. However, the presence of lesions with greater diffuse involvement of the parenchyma, extending to the cortex, is more suggestive of a tumor than demyelination. In addition, reliance on clinical history and manifest clinical signs is necessary for the differential diagnosis. This highlights the importance of performing a biopsy to conclude the diagnosis. Our patient met both diagnostic criteria, classifying her as Type I GC.

There is no established treatment regimen, as GC is nonresectable and surgery only serves a diagnostic purpose. Current evidence-based oncological therapy consists of radiotherapy and chemotherapy [6], but the prognosis is often very poor, as it is an unresectable tumor, the only evidence on the effectiveness of chemotherapy and/or radiotherapy treatment comes from isolated cases, so its impact on survival is contradictory [1]. This patient did not receive oncological therapy due to the family's refusal upon learning of the poor prognosis. She is being treated with dopaminergic drugs, NMDA antagonists, and antipsychotics, with limited success.

## 4. Conclusion

GC remains an infrequent pathology with a wide variety of clinical presentations, leading to delayed diagnosis. Among the broad spectrum of presenting symptoms, parkinsonism symptoms are even less common. Therefore, we found the case of the patient who presented with such symptoms and signs particularly significant. In atypical presentations of parkinsonism, a complete etiological protocol should be performed, considering GC as a possible diagnosis.

The limited results in treating this pathology remain a challenge in research for more effective treatments. Therefore, supportive treatment should be continued as described in most literature studies and as currently administered to our patient.

## Data Availability

The data of clinical record of this patient are available from the author upon request.
